# Serum Interleukin-6 Expression Level and Its Clinical Significance in Patients with Dermatomyositis

**DOI:** 10.1155/2013/717808

**Published:** 2013-09-03

**Authors:** Min Yang, Xiaomin Cen, Qibing Xie, Chuan Zuo, Guixiu Shi, Geng Yin

**Affiliations:** Department of Rheumatology and Immunology, West China Hospital of Sichuan University, Chengdu, Sichuan 610041, China

## Abstract

*Objective*. To analyze serum interleukin-6 (IL-6) expression level and its clinical significance in patients with dermatomyositis. *Methods*. Blood samples from 23 adult patients with dermatomyositis (DM), 22 with systemic lupus erythematosus (SLE), 22 with rheumatoid arthritis (RA), 16 with Sjögren's syndrome (SS), and 20 healthy controls were collected. The IL-6 concentration was detected by chemiluminescence immunoassay. Correlations between IL-6 expression levels and clinical features or laboratory findings in patients with DM were investigated. *Results*. IL-6 expression level of DM patients was significantly higher than that of normal controls, significantly lower than that of RA patients, and slightly lower than that of SLE or SS patients with no significant differences. The incidence of fever was significantly higher in the IL-6 elevated group. Serum ferritin (SF) and C-reactive protein (CRP) were positively correlated with IL-6. *Conclusions*. IL-6 plays a less important role in DM than in RA. IL-6 monoclonal antibodies may have poor effect in patients with DM.

## 1. Introduction

Idiopathic inflammatory myopathy (IIM) is a series of disorders characterized by chronic muscle inflammation of unknown cause. Dermatomyositis (DM) is one of the most common forms of IIM with muscle, skin, and internal organs involved [1, 2]. Interleukin-6 (IL-6) is a potent, pleiotropic Th2 cytokine that regulates the immune defense response. IL-6 acts as both a proinflammatory and an anti-inflammatory cytokine and plays a central role in the transition from the acute to the chronic phase of the inflammatory process [3, 4]. Elevated levels of IL-6 have been documented in a variety of autoimmune diseases, such as rheumatoid arthritis, inflammatory bowel disease, glomerular nephritis, and so forth [5–7]. In this study, we tried to determine whether IL-6 could contribute to the inflammation associated with DM. We compared serum IL-6 level in DM patients with other three connective tissue diseases: systemic lupus erythematosus (SLE), rheumatoid arthritis (RA), and Sjögren's syndrome (SS). Clinical significance of IL-6 was evaluated.

## 2. Materials and Methods

### 2.1. Patients and Controls

All patients were enrolled in West China Hospital from August 2012 to March 2013. Patients with DM satisfied the criteria of Bohan/Peter [8, 9]. The disease controls were patients with SLE, RA, and SS, based on the Criteria of ACR for SLE [10], the Criteria of ACR for RA [11], and the revised American-European Criteria for SS [12], respectively. Patients with an overlapping syndrome were excluded. Healthy controls were matched to DM patients by age and gender. This study was approved by the Ethical Committee of West China Hospital of Sichuan University, and informed consent was obtained from all patients.

### 2.2. Methods

Serum IL-6 levels were measured by chemiluminescence immunoassay (ROCHE Modular Analytics E170) in DM patients and controls. Blood parameters including creatine phosphokinase (CPK), CRP, erythrocyte sedimentation rate (ESR), SF, antinuclear antibody (ANA) and anti-Jo-1 antibody were measured by standard methods. Clinical data were obtained from medical records on admission.

### 2.3. Statistics

Statistical analysis was performed by using Fisher's exact test for comparison of frequencies and Mann-Whitney *U* test for comparison of median levels. Correlation coefficients were established by using Spearman's correlation coefficients. The data were analyzed by SPSS 17.0 software. *P* < 0.05 was considered to be statistically significant.

## 3. Results

### 3.1. Serum IL-6 Levels in DM Patients and Controls

**Figure 1:**
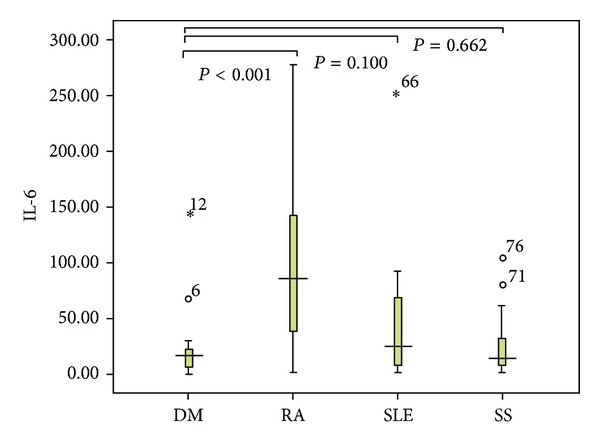
Serum IL-6 levels in patients with DM, RA, SLE, and SS.

Twenty-Three patients with DM, 22 with SLE, 22 with RA, 16 with SS, and 20 healthy controls were enrolled in this study. The demography and the IL-6 levels in DM patients and controls were shown in Table 1 and Figure 1. The age at onset and sex were not significantly different between DM patients and controls. The serum level of IL-6 was 21.5 ± 30.3, 43.3 ± 55.6, 102.6 ± 82.8, 26.7 ± 29.9, and 1.2 ± 2.6 pg/mL in DM, SLE, RA, SS patients, and healthy controls, respectively. The level of IL-6 was significantly higher in DM patients than that in healthy controls (*P* < 0.01), and was significantly lower than that in RA patients (*P* < 0.001). Compared with SLE and SS patients, IL-6 level was slightly lower in DM patients, but no significant difference was found.

### 3.2. Correlation between IL-6 and Clinical Features or Laboratory Markers

**Figure 2:**
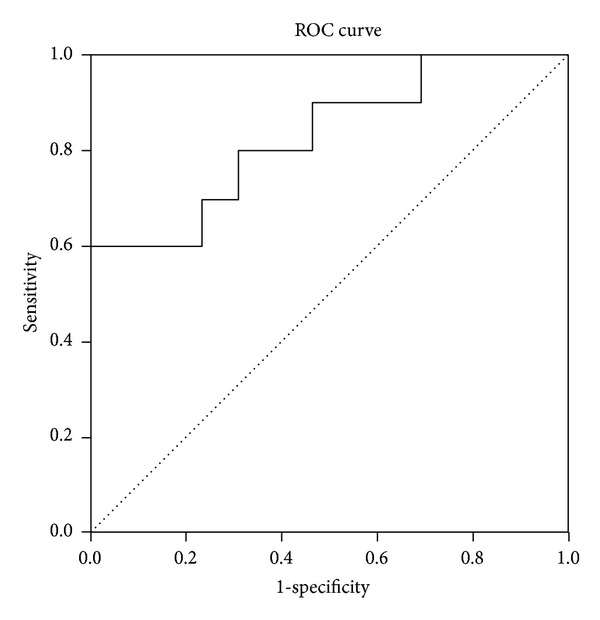
The ROC curve of DM patients with fever.

Correlation coefficients were established by using Spearman's correlation coefficients. Considering the clinical characteristics, significantly positive correlation was found between IL-6 and fever (*r*_*s*_ = 0.569; *P* = 0.005, Table 2). As shown in Figure 2, the receiver-operator characteristic curve (ROC curve) analysis was used to determine the level of IL-6 when fever occurred. The area under the curve was 0.831, and the cutpoint value was 22.35 pg/mL. All DM patients had fever when IL-6 level was above the cutpoint. The sensitivity and specificity for IL-6 cutoff point value predicting fever in DM patients were 60% and 100%, respectively. In addition, no correlation was found between IL-6 and sex, age, course of disease, muscle weakness, interstitial lung disease, Gottron's sign, heliotrope eruption, or arthritis. As for laboratory markers, IL-6 was found positively correlated with CRP (*r*_*s*_ = 0.595; *P* = 0.004) and SF (*r*_*s*_ = 0.789; *P* = 0.004, Table 3). No correlation was found between IL-6 and abnormal electromyogram, CPK, positive muscle biopsy findings, ESR, ANA, or anti-Jo-1.

## 4. Discussion

It is widely accepted that DM arises from CD_4_^+^ T cell- and B cell-mediated muscle inflammation, in which the complement system is activated, resulting in membrane deposition of attack complex within muscle capillaries [13]. It is unclear which pathway mediates the inflammation and perivasculitis [14]. As far as we know, IL-6 is an important proinflammatory cytokine and contributes to the inflammatory process. Elevated levels of IL-6 have been documented in a variety of autoimmune diseases, including rheumatoid arthritis, colitis, Crohn's disease, glomerular nephritis, and so forth [15]. Besides, IL-6 plays central roles in the regulation of both innate and adaptive inflammatory immune responses, as well as both humoral and cell-mediated autoimmune reactions, such as B cell differentiation activity and the T cell growth and differentiation [16]. In literature, evidence concerning the pathogenic role of IL-6 in DM is sparse. Our study demonstrated that the level of serum IL-6 was significantly higher in DM patients than in healthy controls, and IL-6 had significant correlation with the inflammatory marker CRP or serum ferritin (SF). In pathophysiology, tissue injury of skin, muscle, or other internal organs activates monocytes and macrophages to release cytokines, inducing in turn hepatic synthesis of acute phase reactants (APRs) [17]. CRP and SF are classic APRs, and IL-6 is a mainly stimulator of APRs [18]. These findings showed that IL-6 played a role in the inflammatory process of DM. Interestingly, this conclusion was verified in an experimental mouse model of myosin-induced myositis, indicating that the deficiency of IL-6 led to marked amelioration of the clinical signs and pathologic findings of muscle injury [19].

Furthermore, we found that the level of IL-6 in DM patients was significantly lower than that in RA patients, which suggesting a less important role of IL-6 in DM than in RA [20]. The level of IL-6 in DM was even slightly lower than those in SLE and SS. In DM patients, the degree of inflammatory reaction is not always consistent with clinical severity, and inflammation decrescence may be accompanied with aggravated systemic injuries. The effect of anti-inflammatory drugs as glucocorticoid on DM is limited to some degree. These phenomena provided clinical evidence to our deduction that inflammation is only a small chapter in the pathogenesis of DM, and IL-6 plays a minor role in it. Therefore, we can conclude that although blockade of IL-6 and IL-6 signaling have been shown to be effective in treating several inflammatory diseases (e.g., tocilizumab for rheumatoid arthritis and inflammatory bowel disease [21, 22]), its effect in patients with DM should be limited.

RA, SLE, and SS are known to have immune activation, producing a lot of antibodies. However, few DM patients have myositis-specific antibody (MSA) or myositis-associated antibody (MAA) [23]. Autoantibody production relies on B cell differentiation and activation. As mentioned above, IL-6 plays central roles in B cell differentiation activity. Thus, lower serum IL-6 level in DM than in RA, SLE, and SS may be an explanation to the differences in autoantibody production in these rheumatic diseases. In this study, we found that the level of IL-6 was closely related to fever. DM patients whose IL-6 level was above the cutoff point almost had fever. In view of pathology and physiology, IL-6 plays an important role in the mechanism of fever by changing the thermoregulation set-point [24]. It is interesting that IL-6 is significantly higher in RA patients, but fever is seldom seen. The mechanism for this needs further research. 

In conclusion, we believe that IL-6 plays a minor role in DM. Large samples and longitudinal studies are required to elucidate the exact relationship between IL-6 and DM.

## 5. Conclusions

IL-6 plays a less important role in DM than in RA. The effect of the therapy targeting IL-6 on DM may be limited.

## Figures and Tables

**Table 1:** The demography and the IL-6 levels in DM patients and controls.

Variables	DM	SLE	RA	SS	Healthy controls
	*n* = 23	*n* = 22	*n* = 22	*n* = 16	*n* = 20
Age, years	49 ± 11	41 ± 16	53 ± 14	44 ± 14	45 ± 12
Male/female	9/14	1/21	8/14	1/15	10/10
IL-6, pg/mL	21.5 ± 30.3	43.3 ± 55.6	102.6 ± 82.8	26.7 ± 29.9	1.2 ± 2.6
*P* value		0.100	<0.001	0.662	<0.01

*P* value was obtained from the statistical comparisons of serum IL-6 between the DM group and the SLE, RA, SS, and healthy groups. DM: dermatomyositis, SLE: systemic lupus erythematosus, RA: rheumatoid arthritis, SS: Sjögren's syndrome, and IL-6: interleukin-6.

**Table 2:** Correlation between IL-6 and clinical features in patients with DM.

Variables	*r*_*s*_	*P* value
Sex	−0.013	0.952
Age (years)	0.009	0.968
Course of DM (months)	−0.322	0.134
Muscle weakness	−0.011	0.961
ILD	0.100	0.651
Gottron's sign	0.215	0.325
Heliotrope eruption	0.121	0.582
Arthritis	0.079	0.721
Fever	0.569	0.005

ILD: interstitial lung disease.

**Table 3:** Correlation between IL-6 and laboratory markers in patients with DM.

Variables	*r*_*s*_	*P* value
EMG	0.081	0.715
CPK	0.405	0.055
Biopsy	0.228	0.295
ESR	−0.019	0.930
CRP	0.595	0.004
SF	0.789	0.004
ANA	−0.079	0.721
Anti-Jo-1	−0.111	0.613

EMG: electromyogram, CPK: creatine phosphokinase, ESR: erythrocyte sedimentation rate, CRP: C-reactive protein, SF: serum ferritin, and ANA: antinuclear antibody.
